# Transformative Readings: Harry Potter Fan Fiction, Trans/Queer Reader Response, and J. K. Rowling

**DOI:** 10.1007/s10583-021-09446-9

**Published:** 2021-03-28

**Authors:** Jennifer Duggan

**Affiliations:** University of South-Eastern Norway, Grønland 58, 3045 Drammen, Norway

**Keywords:** Fandom, Fan fiction, Gender minority, Harry Potter, Reader response, Trans

## Abstract

The politics of children’s literature and the actors surrounding it have never been more visible than they are now, in the digital age. As one of the first children’s series to gain widespread popularity concurrently with the spread of the internet, the Harry Potter septet arrived on the global stage at the perfect moment to develop an avid, connected fandom. But the fandom has laid bare the many conflicting ideologies of the fans themselves and of the actors surrounding the texts. This article examines the contentious issue of gender nonnormativity and its relation to the Harry Potter texts, the queer/trans reading practices and political resistance common to the fandom, and the ongoing disagreements over gender, made visible on social media, between Rowling and the fans of her series. The article discusses the Harry Potter novels’ varied and conflicting ideologies; queer/trans readings of the Potter septet, including both invitations and resistances to queer/trans reading by Rowling herself; how gender is queered and queried in and through fan fiction; and finally, the recent hostilities between Rowling and her fans. It concludes by discussing the worsening relationship between Rowling and her fans and highlighting how fans are using their collective power to undermine Rowling’s gender politics through fan fiction. By doing so, the article traces the complex politics of the reception of books for young people in the digital age, demonstrating that authors’ powerful voices continue to shape readers’ responses to texts long after their publication but showing, too, that readers often resist authors’ attempts to influence not only their textual interpretations but their politics.

## Introduction

The internet and social media have escalated a shift in reading practices toward participatory, networked communities of interpretation (Jenkins, 2006). As one of the first children’s series to gain widespread popularity concurrently with the spread of the internet, the Harry Potter septet (1997–2007) arrived on the global stage at the perfect moment to develop an avid, connected fandom. Although fans predicted that their beloved fandom would wither after the publication of *The Deathly Hallows* (2007; Tosenberger, 2007, p. 163), the fandom has remained robust and active. Some might argue that this activity is thanks to new texts—the *Fantastic Beasts* (2016–) film franchise is ongoing, and *Harry Potter and the Cursed Child* (2016) has been a hit—but there is more happening in the fandom than meets the eye.

The politics of children’s literature has never been more visible. The Harry Potter fandom, in particular, has been a political battlefield. Many fans feel they are fighting for inclusion and against discrimination, including against a conservative children’s publishing industry, against exclusionary political movements, and against author J. K. Rowling herself (cf. Brough and Shresthova, 2012; Jenkins, 2012). Readers, young and old, are insisting on the primacy of their own interpretations, which often rely on the coalitional politics espoused by Harry and his allies in the septet. Yet others align themselves with exclusionary politics, citing authority figures like Warner Brothers, Rowling, and the admittedly contradictorily heteronormative and white-centered books themselves (Duggan, 2019; Galway, 2012; Horne, 2010; Pugh and Wallace, 2006, 2008; Rana, 2011; Thomas, 2019). These exclusions are particularly apparent when fans exclude either fans or fan-created representations of characters who are members of traditionally marginalized groups (Fowler, 2019; Walton, 2018). Arguments abound between “transformational” and “affirmational” fans, that is, between fans who appropriate texts for their own purposes and those who seek to support or reiterate the author’s perceived intentions (Tosenberger, 2014).

This paper seeks to examine one disagreement that was catapulted into news feeds in the summer of 2020. While fans’ fight for inclusion spans a range of identity markers, including race (Fowler, 2019; Gilliland, 2016; Seymour, 2018; Thomas, 2019) and sexuality (Duggan, 2017, 2019; Fowler, 2019; Tosenberger, 2007, 2008a, b, 2014; Willis, 2006), this article will focus on the contentious issue of gender nonnormativity and its relation to the Harry Potter texts, the queer/trans reading practices and political resistance common to the fandom, and the ongoing verbal sparring match between Rowling and Harry Potter fans, in which numerous fans have critiqued Rowling’s suggestion that trans rights are “ero[ding] … women’s and girl’s rights” and that trans individuals’ presence in toilets and changing rooms is dangerous to women (Rowling, 2020k). The article will first discuss the original seven Harry Potter novels themselves, examining the conflicts between their explicit endorsement of tolerance and their more conservative undercurrents. It will discuss queer/trans readings of the Potter septet, including both invitations and resistances to queer/trans reading by Rowling herself and embedded within the texts, as well as discussing the transformative works produced by fans, focusing on how gender is queered and queried in and through fan fiction. Finally, it will discuss the recent hostilities between Rowling and her fans, considering the role authors play in readers’ interpretations of their texts.

## Harry Potter’s Plurality of Meanings and Contradictory Ideologies

Reader response theory claims the author is “dead” (Barthes, 1977). It tells us that texts come into being somewhere between what is written and the reader, in the reader’s interpretation of the text (Barthes, 1974; Fish, 1980; Iser, 1972). Texts that inspire multiple interpretations, aptly called “writerly” texts, have a “plurality of entrances, … opening[s] of networks, … infinit[ies] of languages” (Barthes, 1974, p. 5). These texts take advantage of the gaps that open up during the “metonymic skids” of reading (Barthes, 1974, p. 92). They deny the text an ultimate meaning and instead allow readers “to appreciate what *plural* constitutes” a text (Barthes, 1974, p. 5, emphasis in original). For Roland Barthes (1974) and Wolfgang Iser (1972), all texts have gaps that invite readers’ interpretive acts. As Iser argues, “no reading can ever exhaust the full potential [of a text], for each reader will fill in the gaps in his own way” (1972, p. 285).

Serial narratives in particular invite “writerly” responses because they invite readers “to enter, interpret, and expand the text” (Hellekson and Busse, 2006, p. 6). Series invite “anticipation and retrospection” or open “up a particular horizon” (Iser, 1972, pp. 285, 283) in which the multiple directions a story could take remain open to conjecture. There are many gaps in the Harry Potter septet that allow readers to respond to the texts, not least the gaps between the books and the gaps created by the almost exclusive focalization through Harry. The intratextuality of the Harry Potter septet is a “repetition as adaption and unfolding, repeating always with a difference” (Dresang and Campana, 2014, p. 96).

More, the Harry Potter novels interweave various genres, including the school story, mystery and detective fiction, fantasy, and Bildungsroman. They not only emphasize the protagonists’ “moral and emotional development” through focalization and the stylistic weighting of “subjective, internal experience” (Westman, 2011, p. 96) but also “desirable models of child behavior and development” (Flanagan, 2014, p. 5; see also Daley-Carey, 2018). While the novels feature as a protagonist a heroic white, wealthy, Western, middle-class, able, cisgendered boy, they nonetheless align that boy with various forms of alterity—as an outsider to the magical community, having been raised by nonmagical “muggles”; as an abused and neglected orphan; as tainted by accusations of madness and an alleged pederastic entanglement with Dumbledore; and as a friend to the poor, the weak, the enslaved, the nonhuman, the tainted, and the abused (Duggan, 2019; Nikolajeva, 2010; Pugh and Wallace, 2006, 2008). This creates a gap between the somewhat conservative traditions shaping the series and the books’ overall message of inclusion, coalition, and resistance to oppression. Indeed, the main thematic focus of the books is that the socially marginalized and their allies can, through coalition, successfully battle prejudicial policies and actions. The contradictory, slippery messages and structures of the septet, combined with the novels’ seriality (Hellekson and Busse, 2006; Dresang and Campana, 2014), open up a number of gaps through which readers can create meaning and around which they can build interpretive communities (Fish, 1980).

Perhaps the most intriguing gaps, which have inspired the most transformative work by readers, are those between the texts’ explicit and implicit ideologies. Ideologies are, of course, present in all literature: “all aspects of textual discourse, from story outcomes to the expressive forms of language, are informed and shaped by ideology[,] … a system of beliefs which a society shares and uses to make sense of the world and which are therefore immanent in the texts produced by that society” (McCallum and Stephens, 2011, p. 360). These ideologies may be considered by readers to maintain or subvert systems of dominance and oppression (McCallum and Stephens, 2011; Van Dijk, 2003). The ideologies encoded in texts are often perceived to be desirable or undesirable based on a reader’s positionality relative to a given text and the society in which it was produced. Readers may even work actively together as a community to oppose certain ideological messages in the text and focus instead on others they find more palatable (cf. Fiske, 2010; Hall, 2001).^1^

As John Stephens argues in *Language and Ideology in Children*’*s Fiction*, children’s texts lie “firmly within the domain of cultural practices which exist for the purpose of socializing their target audience” (1992, p. 8). However, the ways in which ideologies are written into children’s texts differ; some are explicit and didactic, while others are more covert, encoded within the language and imaginative premises of the narrative worlds presented to readers. Stephens identifies three kinds of ideology that are usually present in children’s texts: the first are explicit ideologies, which usually aim to “openly advocate for ‘progressive’ or ‘enlightened’ ideas” (1992, p. 9). He and Robyn McCallum later refer to these ideologies as “topicalized.” Topicalized ideologies are those that appear “as … overt or explicit element[s] in the text, expressing the writer’s social, political, or moral beliefs” (McCallum and Stephens, 2011, pp. 361–362). They are linked to children’s texts’ intent to socialize their readers.

In the Harry Potter texts, the clearest topicalized ideology aligns itself with subjugated minorities and against systems of oppression. The books clearly champion the abnormal over the normal through the contrast of the “perfectly normal” Dursley family with the decidedly less normal wizarding world, as well as through Harry and his friends’ resistance to oppressive politics within the wizarding world (Duggan, 2019; Pugh, 2011; Pugh and Wallace, 2006, 2008). The books explicitly promote acceptance across difference and the emancipation of the oppressed through their rejection of biased courts, corrupt politicians, oppressive teaching practices, pure-blood aristocratic values, the subjugation of sentient magical creatures, discrimination against half-giants and werewolves, and the use of violence, torture, and death as tools of political oppression.

The second type of ideology present in children’s texts is “passive ideology,” that is, “the implicit presence in the text of the writer’s unexamined assumptions” (Stephens, 1992, p. 9) or “assumed social structures and habits of thought … [which affirm] that ‘this is the way things are’” (McCallum and Stephens, 2011, p. 363). For example, in Harry Potter the most visible relationships are those of married, heteronormative, procreative couples. While other kinship structures and ways of desiring may be implied, for example through Remus and Sirius’s giving Harry a joint Christmas present (Tosenberger, 2008a, pp. 196–197), the men are never explicitly described as a couple in the way heteronormative couples are. Similarly, Dumbledore, who was “outed” by Rowling after the publication of *The Deathly Hallows* (2007), is never explicitly described as gay in the septet (e.g., Duggan, 2019; Pugh, 2011; Salter and Stanfill, 2020; Tosenberger, 2008b). The novels thereby subtly suggest to readers that the traditional family is the sole viable way of living, or at very least, the preferable one.

The final type of ideology present in a text is that which is enfolded in the language of the text (McCallum and Stephens, 2011, p. 370; Stephens, 1992, pp. 10–11). This third and final level of ideology is also implicit and is the most subtle of the ideological layers. A pertinent example from the septet is the use of binary gender pronouns throughout. This exclusive use of binary pronouns implies that only two gender identities are available to characters and, by extension, readers.

While the explicit ideology of the Harry Potter series is progressive, the implicit ideologies are, in general, rather conservative. It thus remains up to readers to decide whether the main ideology communicated is positive, negative, or neutral, and to choose whether to focus on the explicit ideology, encouraging a coalitional politics of freedom, or the implicit conservative ideology. The gaps between the explicit and implicit ideologies are also the impetus for many of the academic debates surrounding the septet, regarding, for example, whether or not they can be considered feminist, racist, ableist, speciesist, and so forth. As McCallum and Stephens (2011) argue, readers will attribute different ideological significances to a text informed by their own subject positions, lived experiences, and interpretations or prioritizations of the ideologies within a text.

## Trans and Queer Reading

Through the protagonists’ modelling such behaviors, the Harry Potter septet encourages readers to seek out various sources of information and to make up their own minds about events’ significance, what choices are ethical, and which ideologies to hold. Indeed, the protagonists spend much of their time seeking out information that has been withheld from them. Headmaster Albus Dumbledore also repeatedly tells Harry that the mistakes Harry and his friends have made are in fact due to Dumbledore’s suppression of information he felt would compromise Harry’s presumed innocence, and the texts therefore question a paradigm of child protectionism (Pugh, 2011).

Beyond this, the books encourage readers to interpret a number of gaps, silences, and contradictions. This is in part due to their structure as a series and the influence of the mystery and detective genres on the texts, but it is also due to how Rowling uses pregnant silences as a way to include (while technically avoiding) subject matter not typically seen as appropriate for child readers. As Tison Pugh argues, Rowling regularly “slyly introduce[s] numerous topics typically considered taboo for children – vulgarity, bestiality, alcoholism, excrementality,” and homosexuality (2011, p. 86). The careful occlusion of transgressive topics allows Rowling to introduce them even while they remain absent from the text and to use humor to deflect criticism. Rowling’s tongue-in-cheek, often euphemistic depictions of sexuality, while usually focused on heteronormative entanglements, include cross-species and homosexual hints (Duggan, 2019, pp. 96–101; Pugh, 2011). Reading into these sly silences has been openly encouraged by Rowling, who announced to her fans after the release of *Deathly Hallows* (2007) that Dumbledore was gay and stated that sensitive readers would notice hints of his homosexuality (e.g., EdwardTLC, 2007; HarryPotterAdmirer, 2013).^2^

Such “sensitive reading,” or reading into gaps and silences, closely mirrors what has traditionally been termed “queer reading.” Because queer topics were for so long taboo, queer readers developed strategies that allowed them to see the latent queerness of texts. Although some have suggested that queer reading purposefully ignores the primary meanings of many texts, both Alexander Doty (1993) and Catherine Tosenberger (2008a, b) argue that queer reading is not reading against the grain but rather a lens or practice that encourages readers to pay attention to the subtextual or surface-level queer elements of texts. Frankly, it would be difficult to argue that the Harry Potter texts do not contain queer elements, as a surface-level reading of the first novel quite literally documents Harry’s being freed from life in a closet and introduced to a nonnormative magical subculture by a pink-umbrella-wielding half-giant.

The queerness of the Harry Potter books has been well documented in previous research (e.g., Duggan, 2019; Pugh, 2011; Tosenberger, 2007, 2008a, b, 2014). However, little scholarship has paid close attention to the subtle ways in which characters’ gender performances occasionally undermine hegemonic understandings of binary gender. Moreover, almost no Harry Potter scholarship has taken “into consideration the multifaceted and interactive relationship readers have with a text within a culture and the complexities of shifting, layered, gendered identities that exist with, on the margins of, or in opposition to cultural norms” (Wannamaker, 2008, p. 122). By the term “gender,” I refer to the complex confluence of bodily materiality, felt sense of self, and our own and others’ placement of us within a discursive system. While some feminist, queer, and trans scholars consider sex and gender to be separate categories, with “sex” describing biological or material attributes we have at birth, such as breasts, a penis, hormones, or chromosomes, and “gender” describing the expression or practice of masculinity or femininity, I understand sex always already being gender, following Judith Butler’s (1999, 2004, 2011) argument that sex is always already interpreted through a cultural lens which places the material markers of sex within a medicolegal, sociopolitical, historically contingent binary system.

Annette Wannamaker argues that the Harry Potter series’ representations of gender, while binarized, “depict the anxieties, tensions, and uncertainties about contemporary gender roles that readers of all ages are continuously working to define and negotiate” (2008, p. 122). She emphasizes that the wizarding population in the novels is regularly portrayed as abnormal precisely because of its nonhegemonic gendered behaviors and dress: wizards are often depicted wearing bright or feminine colors, robes that appear dress-like, or unconventional clothing for their apparent genders. Moreover, they are often shown to be more physically and emotionally affectionate than traditional gender roles would allow, for example by hugging strangers on the street. Wannamaker argues that these “abject” depictions of gender:

[open] up gaps that can make [gender] performance visible and create moments of “productive crisis” …. The portrayal of gender in the Harry Potter series is often ambivalent and mirrors … messy, contradictory reality…. It is within these contradictions that spaces open up to view gender … in the novels in alternative ways. (Wannamaker, 2008, pp. 128, 130)

By depicting gender as varied and contradictory, the Harry Potter books leave open spaces in which readers can consider further fluctuations. Indeed, such imaginings are encouraged by the books themselves, which include cross-species attractions, as exemplified by the attraction of numerous female students to the centaur Firenze; mixed-species characters like Hagrid (a half-giant whose father was human and whose mother was a giant) and Fleur (one-quarter Veela); the material transformations made possible by glamors (spells that can change one’s appearance) and potions like the Polyjuice Potion (which allows the drinker to temporarily take on another’s body); and the bodily fluidity of Animagi (human–animal shapeshifters), Metamorphmagi (born shapeshifters who can change their appearance at will), and magical creatures such as werewolves and Veela (who transform under specific stimuli). Although the characters in the novels usually limit their transformations to the gender they were assigned at birth, mishaps such as Hermione’s accidental use of a cat hair in her Polyjuice in the second novel and jokes made by the characters suggest early in the series that the barriers between the genders are just as permeable as those between species. This is confirmed in the two final books in the series, *Half-Blood Prince* (2005) and *Deathly Hallows* (2007): in the former, two male characters, Crabbe and Goyle, transform into young female students multiple times to surreptitiously guard the door to the Room of Requirement for Draco (Rowling, 2005, pp. 270, 386, 396, 425–426), while at the beginning of the latter, Hermione and Fleur, both female, are part of a cohort who magically morph into decoy Harrys (pp. 42–57). It is therefore unsurprising that boundary crossings have made their way into fans’ transformative interpretations of the novels.

## Fannish Transformations

The Harry Potter fan community is extraordinarily large, varied, and productive (e.g., Dresang and Campana, 2014; Jenkins, 2006; Tosenberger 2008a, b, 2014). This is likely in large part due to the gaps in the commercial septet, discussed above, which allow readers to negotiate multiple meanings and to build an “interpretive community” (Fish, 1980), as well as to the timing of the books’ publication, which coincided with the internet’s spread, and the immense popularity of the septet and its film adaptations. Moreover, the online Harry Potter fan community has proven to be particularly attractive to those who feel marginalized by hegemonic culture (Duggan, 2017, 2020, forthcoming; Fowler, 2019; Tosenberger, 2014). One reason this series attracts such fans is made clear above: Harry aligns himself with the disenfranchised. A second is that fan cultures have a long history of being associated with those who are “deviant,” odd, or otherwise occupy the cultural margins (Jenson, 1992). This includes children (Hunting, 2019), who are likely to feel alienated and disempowered in “aetonormative”—or adult-centered—societies, in which children’s literature can be considered “a refined instrument used for centuries to educate, socialize, and oppress a particular social group” but which can, nonetheless, “subvert its own oppressive function” by showing how “established power structures” can be resisted (Nikolajeva, 2010, pp. 8–9). We might consider the Harry Potter series as a prime example of the anti-aetonormative, carnivalesque impulse of children’s texts (Nikolajeva, 2010). While they certainly aim to prescribe “desirable models of child behavior and development” (Flanagan, 2014, p. 5), they also depict a world in which adults are not to be trusted, politicians are petty and easily manipulated, and children must therefore resist adult expectations and rules, grasp power for themselves, and intervene to create a better world. As discussed above, the characters in the texts, who vacillate between being respected insiders and alienated outsiders throughout the series, often demonstrate the sort of critical thinking that fans take up (Booth, 2015). If implied readers are constructed not outside of but with and through a text (Cocks, 2004), and if we accept that Harry Potter celebrates the marginalized, the odd, and the nonnormative, so too must we consider that Harry Potter not only invites marginalized readers and readings but also invites readers to identify as and/or empathize with those who are marginalized.

It is perhaps unsurprising, given what is discussed above, that of the many subversive impulses of the Harry Potter texts that fans have taken up, queer reigns supreme (Duggan, 2017, 2019, forthcoming; Tosenberger, 2008a, b, 2014; Willis, 2006). The Harry Potter texts, for all their surface-level heteronormativity, are nonetheless deeply queer in that they celebrate “the weird, the strange, and the nonnormative” over entrenched power structures and actors (Duggan, 2019, p. 96). Indeed, Nikolajeva explicitly links the carnivalesque empowerment of the fictional child to queer theory, arguing that “adult normativity is subjected to scrutiny even if the adult is still presented as the norm” (2010, p. 10). There are specific markers within children’s texts that commonly support the subversion of adult–child power structures, such as “the use of specific genres (fantasy, adventure, dystopia),” a diverse range of characters, specific types of settings, and “narrative devices such as voice, focalization and subjectivity” (Nikolajeva, 2010, p. 11). Young people’s participation in fandom takes up these themes and reifies them; indeed, the emergence of the online Harry Potter fandom demonstrates the very real ways young people can circumvent adult restrictions to knowledge, content, and participation (Duggan, 2017).

Speculative genres like fantasy also have strong historical links to queer reading and queer readers. The opportunities available for disidentificatory ruptures is one reason that speculative fictions have proven so popular in fandom, for such fictions have long permitted fantasies of gender and sexuality that may be disavowed or impossible to enact or embody in the material world (Duggan, forthcoming; Lothian, 2008, 2017; Woledge, 2005). Tosenberger argues that fan fiction often reifies themes which, while already extant within a source text, were not made explicit because there was “neither room nor desire” (2014, p. 17). Finding these themes is not “a perverse ‘resistance’ to a given text’s presumed heteronormativity but rather ‘an articulation of latent textual elements’” (Tosenberger, 2008b, pp. 200–201). While some might claim that these elements are subtextual, many readers argue that they are surface elements which ought not to be dismissed or ignored and that, consequently, reading queerly is not necessarily resistant but invited (Jones, 2014).

Harry Potter fans’ queer reading practices were further encouraged by the long-lived dedication of media fans to homoerotic fan fiction, known as “slash.” As experienced media fans migrated to the Harry Potter fandom, they brought with them the queer traditions established elsewhere (Hellekson and Busse, 2006; Jenkins, 1992/2013; Tosenberger, 2008b, 2014). And, while fans had been reading queerly long before Rowling announced that she considered Dumbledore to be gay, her announcement of this fact—followed by the comment, “Oh my God, the fan fiction!”—was taken by the Harry Potter fandom as tacit support of their fan works, the majority of which involve queer romantic pairings and interpretations of the novels (Duggan, 2019; Salter and Stanfill, 2020; Tosenberger, 2008a, b).

But while the queer reading and writing of Harry Potter fans has already been discussed widely by scholars (Duggan, 2017, 2019; Tosenberger, 2008a, b, 2014; Willis, 2006), such discussions have usually focused on sexuality rather than gender. I feel it is an important intervention, then, to emphasize the genderqueer and trans^3^ reading practices of fans if we are to understand why Harry Potter fans are so committed to transgender, intersex, genderqueer, and otherwise nonbinary (hereafter, “gender minority”) identifications and the rights of gender minority individuals. It is also important to note that, within queer fandom, fans have an opportunity to “encounter and experiment with alternative modes of discourse, particularly queer discourse” (Tosenberger, 2008a, p. 186). If we accept that reading practices are deeply intertwined with subjectivity, encountering and exploring these discourses can open up previously unavailable identificatory possibilities (Duggan, forthcoming; McInroy and Craig, 2018). As Iser puts it, “the production of the meaning of literary texts … also entails the possibility that we may formulate ourselves” (1972, p. 299).

Stanley Fish argues that “selves are constituted by the ways of thinking and seeing that inhere in social organizations, and if these constituted selves in turn constitute texts according to the same ways,” both the contexts in which readers find themselves and texts impose some limits to the wealth of possible interpretations (1980, p. 336). For Fish, then, “an interpretive community... constitute[s] the objects upon which its members (also simultaneously constituted) can then agree” (1980, p. 338). The act of reading, in this view, is deeply entangled with the act of self-building, and neither texts nor readers can be disentangled from the social, political, and historical contexts in which they find themselves. However, reading communities can work together to open up and explore the multiple, at times disavowed, identificatory possibilities latent within texts (cf. Fiske, 2010; Hall, 2001).

Fan fiction is often “narrowly focused on bodies or character” and importantly focuses “on repetition and combination” in depicting these bodies (Coppa, 2006, p. 229). Because queer, postcolonial, and critical race theorists locate the most radical opportunities to destabilize hurtful and exclusionary categories in the moment of repetition, fan fiction has a radical potential to destabilize categorizations and binaries such as the gender binary not only for “women” and “men” but also for those who occupy its margins, embody its slippages, or repudiate the binary altogether (cf. Butler, 2004; Muñoz, 2013). Fan fiction thus entails an assemblage of infinite resignifications and ruptures that destabilize the possibility of any single identity’s representation of a normativizing ideal. Fan fiction as radical assemblage acts as a call for constant criticality and dis-ease, a radical designification that envisions subjects as multiple, “constructed and contradictory” (Muñoz, 2013, p. 115). Because fan fiction envisions individual characters as various and varied, characters come to embody “a disidentificatory hermeneutic … [that] clears out a space, deterritorializing it and then reoccupying it with queer and black [and otherwise disavowed] bodies,” thereby allowing fans “to discern seams and contradictions” within any depiction (Muñoz, 2013, p. 115) and to revel in the subversive, carnivalesque polyphony these ruptures permit them to create. By allowing fans to center these disavowed bodies—bodies that are explicitly raced, queered, transed, cripped, or speculatively nonhuman—fan fiction allows fans also to recognize their own uncertain desires as well as multiple (dis)identificatory possibilities (Muñoz, 2013). This means that fans are able, perhaps more than other readers, to recognize the internal contradictions and slippages within identity categories, such as gender, race, nationality, or (dis)ability, and to realize, through fan fiction, the radical potentialities of these categories.

This is not to argue that boundary policing and arguments regarding mismatches between an individual’s or group’s ideal of “man” or “British” or “Harry Potter” do not occur within fandom—of course they do, and of course these disagreements can be vitriolic and hurtful (e.g., Fowler, 2019; Thomas, 2019; Walton, 2018). However, it is inevitably difficult for a fan who has read and seen hundreds of different depictions of Draco, Harry, Ginny, or Blaise Zabini and accepted all of them as valid to completely dismiss new emancipatory revisions. When characters are able to move across genres, genders, races, nationalities, (dis)abilities, and relationships, they are revealed to be “neither constructed nor owned, but [to] have... a life of their own not dependent on any original ‘truth’ or ‘source’” (Coppa, 2006, p. 230).

In the Harry Potter fandom, gender-exploratory fantasies include extrapolating that metamorphmagi, or human shapeshifters, could easily shift between or even blend elements of genders; considering how potions, such as Polyjuice, could allow for gender shifting and exploration; or imagining various ways in which gendered bodies could act otherwise, for example, through “genderswap” or “genderfuck” fan fiction—that is, stories which “use a change of sex to explore... the embodiment of gender” (Lothian, 2008; see also McClellan, 2014)—or through tropes like Alpha/Beta/Omega gendering or male pregnancy, known as MPreg.^4^ Such tropes seem to many to be fantasy, but through a transgender lens of interpretation, we can also consider them to reify, within a fantastic framework, the very real disruptions and schisms gender minority bodies represent to Western binary gender schemas. Fan fiction, by allowing “characters … to remain masculine while becoming female bodied, express femininity in male bodies, or have their stories reworked to incorporate a wider spectrum of gender and embodiment” (Lothian, 2008), thus complicates “traditional feminist and queer … understandings of sex and gender [and] also [reflects] contemporary trends … [and] debates about the relationship between biology and gender” (McClellan, 2014). Queer and trans readings of the Harry Potter texts and the fan fiction based on these readings emphasize the “incoherencies in the allegedly stable relations between chromosomal sex, gender and sexual desire,” demonstrate “the impossibility of any ‘natural’ sexuality,” and “question even such apparently unproblematic terms as ‘man’ and ‘woman’” (Jagose, 1996, p. 3). Charlie Ledbetter (2020) therefore argues that fan fiction “enables subjects to acknowledge oppressive political conditions, engage in collational rebellion, and reimagine societal structures of collective liberation.”

That gender variance is becoming more visible and more acceptable both within fan fiction and other media, as well as in many countries and cultures, also means that realistic gender minority bodies are increasingly making their way into fan fiction. Fans have long created “alternative, more diverse and multi-faceted gender narratives by adding transgender characters to the storyworld or rewriting cisgender (main) characters as trans” (Rose, 2018, p. 107). However, these practices have until quite recently been “treated as an afterthought” (Rose, 2018, p. 108) by fan scholars, who have largely focused on female fans and interpreted fandom through a (traditional) feminist lens, making visible the ways in which fan fiction matters to (implied cisgender) women and girls. Academic practice has made certain kinds of women and girls hypervisible, while minimalizing the visibility of others (Banet-Weiser, 2018; Wanzo, 2015). Gender minority fans and their concerns have often been ignored or, at best, treated as a footnote, even while multiple studies have shown that they make up a significant portion of many fandoms (centreoftheselights, 2013; Duggan, 2020) and while others have shown that fandom is a particularly important arena through which young people come to understand their own and others’ non(hetero)normative sexualities and genders (Duggan, forthcoming; McInroy and Craig, 2018).

Although sexuality has always been the primary concern of scholarship on slash, gender has also been a topic of discussion—from an assertion that slash demonstrates the expansiveness of the category “woman” and women’s sexualities to the recent challenge by gay men (Brennan, 2014; Coleman, 2019) and, increasingly, gender minority fans (Duggan, forthcoming; Ledbetter, 2020; Rose, 2018, 2020) to scholars’ insistence that slash is a women’s genre. Ika Willis argues that slash is “a postpornographic technology of gender involving and enabling a transformation of ‘the body one feels oneself to have’ … [and opening up] certain fantasmatic, identificatory and bodily practices … [that] cut across (*trans-*) [and destabilize] existing categories for sexuality and gender” (2016, p. 290, emphasis in original). She argues that through slash, “the body is experienced – *felt* – differently…. [This] allows us to change our embodiments (the bodies we feel ourselves to have) without changing our physical or corporeal bodies, although some slash fans may (and do) go on to do this” (2016, p. 292). Willis’s focus on cis women in her piece does not diminish the importance of her argument when it is considered in conjunction with a number of recent articles exploring the importance of slash to gender minority fans (Duggan, forthcoming; Ledbetter, 2020; Rose, 2018, 2020). As Willis argues regarding herself, slash can allow fans to think their genders differently. Alexis Lothian argues that “the writing and reading of fans’ sexual fiction is … a [queer] world-making practice” (2017, p. 239), and this practice “opens up a set of possibilities for interrogation, solidification, resistance, destabilization and reconfiguration of gender” and sexuality (Willis, 2016, p. 291), as well as new horizons of identification (Duggan, forthcoming; Rose, 2020).

While “the demographics within slash [communities] remain a constant debate” (Coleman, 2019, p. 91), most scholars agree that these communities are dominated by straight cis women. However, queer and gender minority individuals, who have long occupied the margins of these communities, are increasingly visible (Duggan, 2020). For these fans, slash functions “beyond pleasure, encompassing identity construction and meaning as well” (Brennan, 2014, p. 364). For example, McInroy and Craig’s comparative study of fandom-participating and non-fandom-participating queer youth shows that participation in fandom can expedite queer identity development and that fans use “more non-traditional sexual identity and/or gender identity labels” than non-fans, with a “significant proportion [of fans] … identifying as transgender and/or in some way gender non-conforming” (2018, p. 193). Descriptions of the importance of fan fiction to gender minority individuals include Jackson Bird’s (2019a, 2019b) autoethnographic discussions of how important Harry Potter fandom was to his transition, Jonathan Rose’s (2018, 2020) explorations of transfic as self-narrative, Ledbetter’s (2020) theorization of fan fiction as an expression of political dysphoria, and my recent study demonstrating how slash opens up spaces for gender exploration for gender-minority individuals (Duggan, forthcoming).

Even for those fans who do not consider themselves to be gender minority, it is clear that fan fiction and the communities surrounding it not only emphasize the variance inherent in binarized conceptions of gender but also make visible the instabilities of the binary system (Driscoll, 2006a; Willis, 2016). Because the topicalized ideology of the Harry Potter texts valorizes nonnormativity, and because the depictions of gender in the texts are varied, contradictory, and slippery (Pugh, 2011; Wannamaker, 2008), these texts are particularly attractive to those who disidentify with binarized notions of gender.

We can consider fan works—which are always interpretive, whether they are affirmational or transformational—to include both elements of difference and elements of sameness (Dresang and Campana, 2014; Hellekson and Busse, 2006; Tosenberger, 2014). These works, too, invite interpretations, and as such, encourage readers to recognize the multiplicitous meanings contained within the original texts as well as the variety of interpretations available. Tisha Turk argues that “fan works … redefine both the boundaries of texts and the relationships between creators and audiences… [demonstrating that] the boundaries of the source text’s fictional world are not fixed; rather, they are infinitely expandable” (2011, p. 87). So, too, do they redefine gender identities as unfixed, shifting, and expandable.

## The “Undead” Author: When Readers’ and Author’s Interpretations Collide

Thus far, I have examined the Harry Potter novels themselves and readers’ responses to them, but I would now like to return to their author, J. K. Rowling. I am particularly interested in the position she occupies, the weight her opinions hold, and the effects these have on the reception of her novels and her relationship with her fans. There has been a recent return to considerations of authors and their effects on the reception of texts within the fields of children’s literature and popular culture (e.g., Coats, 2017; Gray and Johnson, 2013). In her overview of debates regarding the position of authors and their influence over textual interpretation, Kristina Busse argues that authors, their identities, and their ideologies are particularly important to “marginal subjects, that is, those who do not occupy upper middle class, white, male, straight, able-bodied, cisgendered, Western positions” (2013, p. 54). Increasingly, readers are holding authors “responsible for hinting at and alluding to queerness without ever delivering” (Nordin, 2019, p. 28). There exist significant overlaps, then, between readers for whom authors’ identifications matter and readers who are likely to become fans.

Renewed scholarly interest in authors has resulted in theorizations of authors as “auteurs” (Busse, 2013; Salter and Stanfill, 2020; Scott, 2013) and “undead” authors (Scott, 2013). Such authors haunt their texts, acting as particularly privileged fans and providing extensions and “authorized” interpretations of their text(s). Rowling is a perfect example of such an author, for she both “refuses to die,” regularly providing increasingly unwelcome extratextual commentary on her own texts, and continues to attempt to control other readers’ interpretations of her texts (Salter and Stanfill, 2020, p. 50; see also Tosenberger, 2008b). Indeed, Rowling’s relationship with the Harry Potter fandom has long been contradictory. On the one hand, she enjoys a “deep and longstanding relationship with fandom … as one of the first authors to welcome fan fiction, engage fans, and fuel the community with direct communication even before social media” (Salter and Stanfill, 2020, p. 40). Of particular interest to this paper is her overt encouragement of both queer readings of the texts and fan fiction (Duggan, 2019; Salter and Stanfill, 2020; Tosenberger, 2008b). On the other hand, she and Warner Brothers have long sought to maintain control over the fandom, fans’ activities, and fans’ interpretations of her texts Rowling not only models “what she sees as correct fandom” (Salter and Stanfill, 2020, p. 40) but actively punishes—both socially and financially—those whose actions are considered “incorrect” (Driscoll, 2006b; Fazekas, 2014; Jenkins, 2006; McLelland, 2011; Salter and Stanfill, 2020). Rowling’s encouragement of fans is an encouragement that extends only to those interpretations she considers to affirm her own vision and to align with her politics, which are less progressive than many fans had initially assumed (Salter and Stanfill, 2020).

Within all fandoms, affirmational behaviors—that is, behaviors that seek to support what is seen to be the author's intended meaning—have long been aligned with those fans who receive societal privilege. For obvious reasons, those who occupy marginalized identities, and who thus privilege counternarratives, reading between the lines, unsanctioned interpretations, “bending,” and multiplicity, are more likely to engage in transformational behaviors, which are less likely to be sanctioned by authors and copyright holders (Scott, 2013; Thomas, 2019). Even though Rowling has been willing to encourage queer interpretations of her texts, her patience is clearly limited to those queer readings she sees as affirming her own interpretations of the text and of what “queer” entails (Salter and Stanfill, 2020). And while fans’ queer reading practices extend to gender nonnormative interpretations, Rowling’s personal, conservative views on sex and gender have recently been made abundantly clear through her repeated and escalating anti-trans commentary, posted between 2017 and 2020 (Jeni, 2017; Rowling, 2019, 2020a, 2020b, 2020c, 2020d, 2020e, 2020f, 2020g, 2020h, 2020i, 2020j, 2020k). To summarize, in 2017, Rowling shared an article critiquing a proposed change to the United Kingdom’s Gender Recognition Act (2004), which was interpreted by some commentators as a change that would allow trans women to access women’s spaces, such as bathrooms (Jeni, 2017).^5^ She then liked and shared a tweet by Janice Turner stating, “No fox has a right to live in a henhouse, even if he identifies as a hen” (Smith, 2019), as well as a post implying that trans women are “men in dresses” (Smith, 2019), and garnered critique for following Magdalen Berns, who insisted that all “trans women are men” (2019) and that “blackface and ‘womenface’ are the same” (2018) on Twitter (see also Rowling, 2020k). The most recent clash between Rowling, the Harry Potter fandom, and trans and queer activists was sparked by Rowling’s (2020a) tweeting in response to an article discussing the health, hygiene, and safety of “people who menstruate” during and after pandemic lockdowns. Rowling (2020a) apparently took issue with the use of the term “people who menstruate” and the article’s explicit inclusion of gender minority individuals (including trans men) in this category, stating, “ ‘People who menstruate.’ I’m sure there used to be a word for those people. Someone help me out. Wumben? Wimpund? Woomud?” The post caused a furor, and Rowling was quick to defend herself with posts claiming that biological sex is “real” (Rowling, 2020b), that she has read widely on subjects relating to sex and gender (Rowling, 2020c), that she supports trans people (Rowling, 2020h, 2020i), that she is being targeted and threatened because she is a woman (Rowling, 2020g), and that the gay and lesbian community feel threatened by gender minority individuals (Rowling, 2020d, 2020j). Finally, she posted a long and, at times, contradictory defense of her position on her blog (Rowling, 2020k).^6^

In this blog post, she not only suggests that trans individuals are a threat to women and that trans-positive discourses are dangerous to children but also that she herself is a victim—in particular, of “accusations of TERF-ery,” threats, and intimidation (Rowling, 2020k). Most alarmingly, she aligns female-to-male transition and trans activism with misogyny and suggests that trans women will be a danger to “biological women” if they are allowed in women’s bathrooms and changing rooms. She uses her history of “domestic abuse and sexual assault” at the hands of a cis male spouse to do so (Rowling, 2020k). This rhetorical use of her past to frame trans individuals as dangerous to women is particularly ironic in light of her firm dismissal of accusations of spousal abuse made against actor Johnny Depp, who stars in the *Fantastic Beasts* (2016–) films, as a private matter (Salter and Stanfill, 2020).

Rowling’s commentary has justifiably been received with horror by many Harry Potter fans, who have learned through her books and the fandom not only to privilege inclusivity but quite specifically to consider gender as expansive and fluid (Bird, 2019a; Coulter, 2018; Pocock, 2019). A great number of fans, as well as the actors involved in the film adaptations of the series (e.g., Lang, 2020; Radcliffe, 2020; Watson, 2020), emphasized in their numerous responses to Rowling’s tweets that gender minority individuals are more vulnerable to sexual violence than others and that the views she has espoused are harmful, hurtful, and exclusionary, particularly to the myriad gender minority and otherwise queer Harry Potter fans for whom the books and fandom have been meaningful spaces of self-discovery. Many commentators have suggested that the Harry Potter series and its fandom were helpful either to them or to friends or family members who were struggling with their gender identities. For example, in an opinion piece in the *New York Times*, trans activist and author Jackson Bird wrote movingly about how the Harry Potter septet allowed him and his fellow readers to learn “the power not just of tolerance, but fierce acceptance and unconditional love” (2019a). He linked this impetus in the text to the “loving, passionate,” and accepting Harry Potter fan community, “a community that continues to foster that same safe space for every queer or trans person who needs it, and which commits itself intentionally toward growth and learning in its inclusion” (2019a). Moreover, Bird argues, “While I was nervous about coming out to some relatives and acquaintances, I never doubted that the Harry Potter fan community would accept me for who I was” (2019a), a sentiment which echoes his earlier writings on the importance of Harry Potter to his personal journey (2019b).

The ongoing conflict between Rowling and many of her fans emphasizes that children’s culture is neither limited by “top-down forces of ideological and institutional control, nor … a free space of individual expression” but is rather “a site of conflicting values, goals, and expectations” (Jenkins, 1998, p. 4). Gender identity remains a “highly conflictual terrain” which is at times “paradoxical” (Willis, 2016, p. 305). Online cultures are important spaces in which trans/queer individuals can explore issues relating to gender/sexuality and open up spaces, modes, and categories in and through which individuals can represent themselves and make themselves visible (Duggan, forthcoming; Ledbetter, 2020; Rose, 2018, 2020) and explore possibilities by “creatively appropriating or exploiting the incoherencies of the binary gender system” (Willis, 2016, p. 305). The rising visibility of trans/queer bodies in fan fiction reflects an increase in the number of fans using labels such as trans, genderqueer, genderfluid, or agender to describe themselves (McInroy and Craig, 2018), demonstrating that influences on identity flow both ways, from the digital to the analogue and back again (Duggan, forthcoming; Ledbetter, 2020; Rose, 2018, 2020). Fan fiction and the digital communities surrounding it allow trans/queer to be perceived as multiple and as opportunities rather than as problematic (Duggan, forthcoming; Rose, 2020), as well as permitting trans/queer “to become more visible and more diverse,” and “transing” to be seen as “a way of engaging with genders and texts, regardless of one’s own gender [identifications and embodiments]” (Rose, 2020, p. 34).

Gender minority individuals report that a key factor that increases acts of discrimination and violence in their day-to-day lives is “behavior by politicians, public figures, [and] community leaders” (FRA, 2020, p. 3). Arguments like Rowling’s (2019, 2020a, 2020b, 2020c, 2020d, 2020e, 2020f, 2020g, 2020h, 2020i, 2020j, 2020k; see also Bird, 2019a; Coulter, 2018; Pocock, 2019; Smith, 2019), which seek to reify discursive categories that are innately unstable, both invisibilize gender-varied bodies and hypervisibilize false notions of binarized sex, which underpin conservative arguments characterizing sex/gender categorization as “real” and “true” as well as invoking harassment, discrimination, and even violence against gender minorities. The war of words between Rowling and her supporters, on one side, and gender minority individuals and their supporters, on the other, demonstrates the fractured feminisms of the current moment, in which more popular, marketable versions of empowered, neoliberal womanhood are both most visible (Banet-Weiser, 2018) and depicted as threatened by those who are marginalized (Phipps, 2016). As Alison Phipps argues, “Disclosing one’s experience of violence in a bid to construct and exclude the Other is violence itself. Especially when personal stories become capital in political debates, they must be understood in relation to dynamics of privilege and marginality” (2016, p. 315).

Rowling’s attempts to silence and dismiss her critics demonstrate the uneven balance of power between authors and readers. As is shown above, Rowling has spent considerable time and effort cultivating a specific type of fandom—one that bows to her interpretive whims—and quashing dissenting voices and movements. The fallout of her most recent “feminist” rants on Twitter and her blog demonstrate just how slippery, contradictory, and contingent readers’ interpretations of the ideologies within and surrounding texts can be. As Busse argues, “Contemporary readers may dismiss authorial intentions but nevertheless rely on authorial identity in their readings and public utterances” (2013, p. 50). Rowling’s carefully cultivated persona as a fan-positive, progressive author is beginning to crack. This is not only because she is receiving justifiable criticism for “apparently wanting credit for things she failed to actually do in her books” (Salter and Stanfill, 2020, p. 46) but also because the very visible auteur persona she has crafted online has revealed her to be much less progressive than the topicalized ideology of the Harry Potter septet implies she is.

Nonetheless, in a world in which gender minority individuals are feeling increasingly discriminated against (FRA, 2020), fan fiction continues to offer utopic visions of what “collective fantasy” could allow (Lothian, 2017, p. 248)—a just and inclusive world created by a coalition of minoritized actors whose experiences of majority-wins politics are “dysphoric” (Ledbetter, 2020). Harry Potter’s “undead” author may refuse to stop shouting from beyond the grave, but readers still hold some collective power. Within the fandom, many fans have openly dismissed Rowling’s perspective, stating their solidarity with gender minority individuals or coming out to their fellow fans in an act of defiance. They have declared that Harry Potter is “theirs” now, to do with what they will—and what many want is to play with gender.

## Footnotes

1 I find the concept of decoding helpful even while I find the idea of a dominant, intended textual ideology problematic. Texts include multiple and contradictory ideologies shaped by the language used, the author’s conscious and unconscious beliefs, the influence of other actors such as editors, the genres and traditions shaping the text, and so forth.

2 On the shifting perception of this announcement, see Duggan (2019), Salter and Stanfill (2020) and Tosenberger (2008b).

3 Because “queer” has come to stand for nonnormativity in general and because it has been criticized for overemphasizing sexuality and marginalizing gender, I emphasize gender-focused queer reading practices, known as “trans reading” or “transing.” See Yep (2013).

4 See Ingram-Waters (2015), Popova (2017), or Weisser (2019) for further information on MPreg and Alpha//Beta/Omega.

5 The proposed changes to the Gender Recognition Act intended to make it easier and less expensive to obtain a Gender Recognition Certificate.

6 There are many further critiques that could be made about Rowling’s stance than this article allows. See, e.g., Alona Ferber’s interview of Judith Butler in *New Statesman* (Ferber, 2020) or the Mermaids UK (2020) “Call to J.K. Rowling” on their website (mermaids_author, 2020).
